# Isolation and Characterization of Plant Growth-Promoting Bacteria from the Rhizosphere of Medicinal and Aromatic Plant *Minthostachys verticillata*

**DOI:** 10.3390/plants13152062

**Published:** 2024-07-26

**Authors:** Romina del Valle Meneguzzi, Marilina Fernandez, Lorena del Rosario Cappellari, Walter Giordano, Erika Banchio

**Affiliations:** INBIAS Instituto de Biotecnología Ambiental y Salud, CONICET-Universidad Nacional de Río Cuarto, Río Cuarto 5800, Argentina; rmeneguzzi@exa.unrc.edu.ar (R.d.V.M.); mfernandez@exa.unrc.edu.ar (M.F.); lcappellari@exa.unrc.edu.ar (L.d.R.C.); wgiordano@exa.unrc.edu.ar (W.G.)

**Keywords:** native rhizobacteria, PGPR, genotyping, peperina, *Stutzerimonas stutzeri*, *Pseudomonas*

## Abstract

This study aimed to isolate and characterize *Pseudomonas* native strains from the rhizospheric soil of *Minthostachys verticillata* plants to evaluate their potential as plant growth-promoting rhizobacteria (PGPR). A total of 22 bacterial isolates were obtained and subjected to various biochemical tests, as well as assessments of plant growth-promoting traits such as phosphate solubilization, hydrogen cyanide production, biocontrol properties through antibiosis, and indole acetic production. Genotypic analysis via 16S rRNA gene sequencing and phylogenetic tree construction identified the strains, with one particular strain named SM 33 showing significant growth-promoting effects on *M. verticillata* seedlings. This strain, SM 33, showed high similarity to *Stutzerimonas stutzeri* based on 16S rRNA gene sequencing and notably increased both shoot fresh weight and root dry weight of the plants. These findings underscore the potential application of native *Pseudomonas* strains in enhancing plant growth and health, offering promising avenues for sustainable agricultural practices.

## 1. Introduction

*Minthostachys* (Benth.) Spach (Lamiaceae) comprises 17 species native to South America, found across the Andes from Venezuela to Argentina. It is a significant genus in Andean traditional medicine and commerce [1]. *Minthostachys verticillata* (Griseb.) Epling, also known as ‘peperina’, is a perennial, gynodioecious semishrub unique to Argentina [1,2]. Peperina is the most commonly utilized species in traditional medicine by local communities [3,4]. Additionally, it is commercially used in the production of liqueurs, bitter drinks, and aromatic mate (a traditional South American infusion) [3,4,5,6,7]. 

Peperina is a species of high conservation priority in central Argentina [3,8] because it is one of the species under the greatest collection pressure. This is evidenced by the decline, weakening in terms of number and density, or disappearance of populations in numerous localities [3,6]. This situation is due to urbanization, collection practices that involve uprooting or not allowing the plants to seed, grazing in these areas, and fires that devastate the vegetation [3,6]. This also leads to a loss of genetic diversity in medicinal and aromatic plants [7]. Productive crops involving peperina are currently scarce, and wild populations of this plant are becoming depleted, putting the species at risk. Meanwhile, companies that demand this aromatic plant—mostly yerba mate producers—require increasingly larger quantities of high-quality raw material [2].

On the other hand, it is important to highlight that for several years, various properties of this aromatic plant have been studied, such as its antibacterial, antiviral, and antiseptic capabilities through its essential oils (EOs), obtaining encouraging results [9,10,11,12,13]. It has been proven that peperina EOs can be used safely and are free from any toxic risk [14]. The capability of these EOs was evaluated against aflatoxin B_1_-induced damage in rats [13] and the antimicrobial power of the EO [15]. It has been demonstrated that peperina EO and one of its pure compounds (limonene) have antimicrobial activity, inhibiting the growth and biofilm formation of pathogens causing bovine mastitis [16]. 

Soils are complex and constantly changing environments with a delicate balance of biological, organic, and mineral components. This reflects intricate connections involving nourishment, coexistence, decay, decomposition, and recycling. The rhizosphere is the part of the soil influenced by the presence of living plant roots [17,18]. Plant roots release various substances such as sugars, amino acids, organic acids, phenolic compounds, vitamins, proteins, and polysaccharides through processes like secretion, exudation, and autolysis of old roots. This increases local nutrient levels, leading to the concentration and assembly of microorganisms in the rhizosphere through chemotaxis and biofilm formation [19,20]. Microorganisms and roots form distinct communities where interaction and communication processes are tightly regulated, controlling or modifying the activities of the entire community [21,22,23,24]. The interacting organisms produce species-specific molecular signals that alter their defense mechanisms and tend to maintain positive relationships among the organisms.

Plant growth-promoting rhizobacteria (PGPR) refers to a group of bacteria that live in the rhizosphere of plants and aid in plant development through various direct and indirect mechanisms [25,26,27,28,29,30]. These rhizobacteria produce growth-regulating substances such as gibberellins, cytokinins, and auxins, which promote the growth of root hairs, increasing the number, length, and surface area of plant roots. This enhances the plant’s ability to absorb water and nutrients, making it more resilient and productive and better able to withstand adverse weather conditions [26,31]. Additionally, these microorganisms help in the solubilization of less mobile soil nutrients, such as phosphorus, which is the second most important nutrient for crop growth after nitrogen. They also contribute to the natural control of plant diseases by boosting the plant’s defense system, thus enhancing its resistance to diseases [28,31].

Among these organisms, *Pseudomonas* is thought to be the most promising group of rhizobacteria that promote plant growth and help control plant diseases [32]. Various *Pseudomonas* strains have several ecologically beneficial traits, such as forming biofilms, producing antifungal substances, mediating quorum sensing, interacting synergistically with plant roots, mediating chemotaxis, and utilizing various plant secretions. *Pseudomonas* species are generally known for promoting plant growth (both roots and shoots) at the molecular level and providing a range of benefits to plants. Multiple strains of *Pseudomonas* contribute to plant health by enhancing growth and biologically suppressing diseases, such as *Fusarium* wilts in wheat and root rot in tobacco [32,33,34]. Particularly, the genus *Stutzerimonas*, previously classified within the genus *Pseudomonas*, is known for its significant plant growth-promoting effects [35]. *Stutzerimonas stutzeri*, in particular, exhibits capabilities such as phosphate solubilization, production of indole-3-acetic acid (IAA), and biocontrol of pathogens [36]. 

The cultivation of native plants for commercial use is currently not common. To successfully grow these plants, it is essential to understand the soil and climate conditions they require [5]. It is also important to improve our understanding of the rhizobacteria that are associated with the plant’s root systems. The rhizosphere plays a significant role in promoting plant health and growth by aiding in nutrient absorption, tolerating environmental stress, and protecting against plant diseases [28]. Therefore, rhizobacteria can be a valuable resource for supporting the cultivation of these plant species. Additionally, identifying and studying rhizobacteria from mountainous soils that have characteristics promoting plant growth from untapped sources can have many practical applications [27,37,38].

The objectives of this study were to isolate, identify, and characterize PGPR strains from the rhizosphere of *M. verticillata*. The study also aimed to evaluate the beneficial effects of these strains on plant growth-promoting properties. Additionally, the study analyzed the phylogenetic relationships based on 16S rRNA sequences of the isolated strains and compared the rhizosphere of *M. verticillata* with the rhizosphere of another aromatic plant: *Mentha piperita.* This comparison was chosen to understand the microbial diversity and potential plant growth-promoting activities in the rhizosphere of these aromatic plants, which are known for their medicinal and aromatic properties.

## 2. Results and Discussion

### 2.1. Physico-Chemical Soil Characteristics

The analysis reveals that the soil possesses a loamy-clay texture with a high clay content (sand: 50–500 µm, silt: 2–50 µm, clay: 2 µm). Table 1 shows a significant amount of organic matter (5.48%) compared to other agricultural soils in the region, indicating excellent soil fertility. Organic matter is crucial as it improves soil structure, water retention, and nutrient supply. The soil also has high levels of phosphorus and nitrogen (23.30 ppm N-NO_3_ and 103.22 ppm NO_3_), which are essential for plant growth. High phosphorus levels promote root development and flowering, while elevated nitrogen levels support vigorous vegetative growth. The excellent water retention capacity (32.10%) is beneficial for crops, particularly in regions with variable rainfall, ensuring that plants have a steady water supply. The near-neutral pH of 7.50 is ideal for most crops, as it optimizes the availability of essential nutrients and minimizes the risk of toxicities associated with very acidic or alkaline soils. The soil’s balanced base ratio and high Cation Exchange Capacity (CEC) of 27.83 cmol/kg, resulting from its substantial clay and organic matter content, enhance its ability to retain and supply essential cations like calcium (16.00 cmol/kg), magnesium (6.50 cmol/kg), sodium (0.90 cmol/kg), and potassium (2.31 cmol/kg). High CEC values indicate that the soil can hold and exchange a large amount of nutrients, which is beneficial for long-term fertility and reduces the risk of nutrient leaching. Additionally, the calcium and magnesium levels are adequate to support plant structure and enzyme function, while the potassium content is sufficient to enhance water uptake and resistance to diseases. Sodium levels are low, which is advantageous as high sodium can lead to soil dispersion and poor structure. Overall, these findings suggest that the soil has significant nutrient and water retention capabilities, making it highly suitable for crop growth and development [39].

### 2.2. Strains Isolatated form M. verticillata Rhizosphere

The isolation of native strains involved collecting rhizospheric soil from spontaneously grown *M. verticillata*, with a total bacteria count of 2.7 × 10^6^ CFU/g. Among the microorganisms that inhabit the soil, bacteria, including actinomycetes, are the most abundant, as they are found in a range of approximately 10^6^ to 10^8^ cells per gram of soil [40]. This finding aligns with previous studies examining rhizospheric soil from *Mentha piperita* commercial crops grown in the San Javier department, Córdoba province, Argentina, where a total bacteria count of 3.05 × 10^7^ CFU/g was reported [41]. Similar bacterial counts have been observed in other aromatic plants, such as *Ocimum basilicum* (0.75 × 10^6^ CFU/g), *Melissa officinalis* (10.00 × 10^6^ CFU/g), and *Origanum syriacum* (20.0 × 10^6^ CFU/g), all grown in loam sandy soil in Egypt [42].

The microbial populations in the rhizospheric soils of different medicinal and aromatic plant species seem to have similar levels of bacterial abundance despite potential differences in environmental conditions and management practices. This consistency highlights the importance of rhizospheric microbiota in aromatic medicinal plants and emphasizes their potential role in plant health and productivity. It is important to note that the number of bacteria in the rhizosphere depends on various factors such as season, soil type, vegetation, moisture content, tillage, and fertilization [43]. However, plants clearly have a significant effect on microbial communities. The diverse microbial communities found in different types of soil used for crop production are influenced by the introduction of crop plants, which can either enhance or suppress the growth of native bacteria [44,45]. There is an ongoing debate about whether soil properties or the introduction of plants primarily determine the structure of microbial communities. It is evident that plants have a significant impact on microbial communities through the composition of their root exudates, which is closely linked to their nutritional status and growth stage [44].

### 2.3. Phenotypic Characterization

Forty-four colonies were randomly isolated from the highest dilution plate counts, representing the most abundant bacterial members of each rhizospheric community. From these, 22 bacterial isolates from the M. verticillata rhizosphere were selected for testing. These isolates were chosen for their Gram-negative characteristics with the goal of identifying native strains of the genus *Pseudomonas*. *Pseudomonas* is the most extensively researched of the known PGPR genera. It has been widely studied due to its wide distribution in various environments and its ease of cultivation under laboratory conditions [46,47]. The collected isolates strains displayed various biochemical activities. Out of the 22 *Pseudomonas* isolates, 8 (36.36%) showed the ability to hydrolyze casein, 2 (9.09%) hydrolyzed lecithin, 2 (9.09%) hydrolyzed starch, and 3 (13.63%) hydrolyzed lipids. Additionally, 8 (36.36%) strains were able to tolerate saline concentrations of 5%, and 10 (45.45%) could tolerate low temperatures (Table 2).

**Figure 1:**
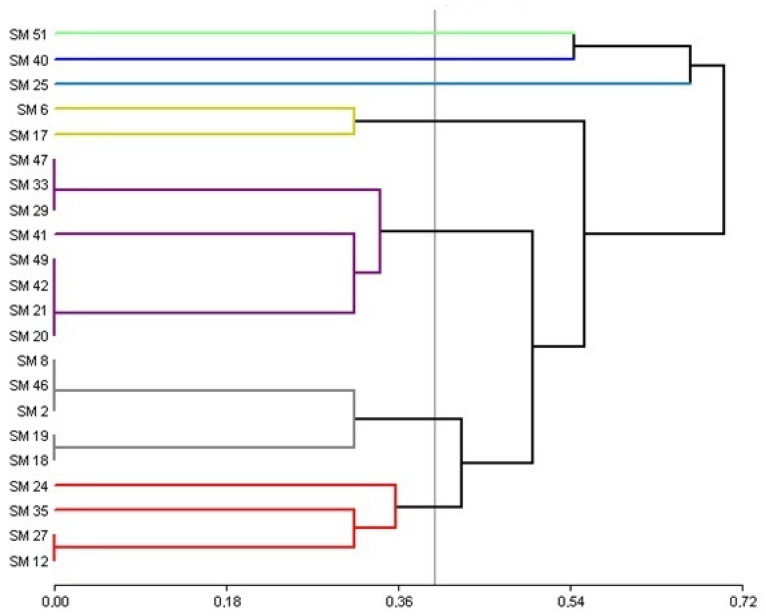
Cluster analysis constructed with variables resulting from the biochemical characterization of *Pseudomonas* strains isolated from the *M. verticillata* rhizosphere.

A multivariate cluster analysis was performed to categorize the strains isolated from the *M. verticillata* rhizosphere based on their similar biochemical characteristics. Figure 1 illustrates the relationships between the strains, providing a better understanding of the variability present in the *M. verticillata* rhizosphere. The analysis grouped the strains into seven categories with similar biochemical profiles. One group consisted of strains SM 20, 21, 29, 33, 41, 42, 47, and 49, characterized by their ability to grow at low temperatures, with 5 of them capable of hydrolyzing casein. The second group, comprising strains SM 2, 8, 18, 19, and 46, was characterized by their growth in 5% saline concentrations. The third group included strains 12, 24, 27, and 35, identified as not exhibiting hydrolytic activity. The fourth group contained strains SM 6 and 17, which were able to hydrolyze casein and lipids. The fifth and sixth groups consisted of strains 25, 40, and 51, known for their growth at high temperatures, with strains SM 40 and 51 also capable of hydrolyzing starch and lipids.

### 2.4. In Vitro Characterization of PGPR Strains

Based on the obtained results, potential mechanisms for enhancing plant growth were identified in the native strains isolated from the *M. verticillata* rhizosphere. As shown in Table 3, out of the 22 isolated strains, 60% exhibited the ability to solubilize phosphates and 50% to produce IAA. The production of IAA is a crucial trait of bacteria that promotes plant growth. It is estimated that up to 80% of microorganisms in soil communities are capable of synthesizing IAA, although the amount of hormone produced may vary depending on the microbial isolate [48]. The studied strains did not demonstrate the ability to produce siderophores. As for biocontrol potential through antibiosis, around 50% of the strains inhibited the growth of the phytopathogenic fungus *Sclerotium rolfsii* by 19 to 50%. *S. rolfsii* was utilized in these tests because it severely impacts many economically important crops, such as legumes, vegetables, and ornamentals, by causing southern blight, which results in significant global yield losses [49].

A multivariate correspondence analysis was performed to reveal patterns and relationships among the PGPR activity in native strains isolated from the *M. verticillata* rhizosphere. This analysis will offer valuable insights into the characteristics and similarities among different strains, leveraging the qualitative data obtained from the PGPR characterization of isolated strains.

**Figure 2:**
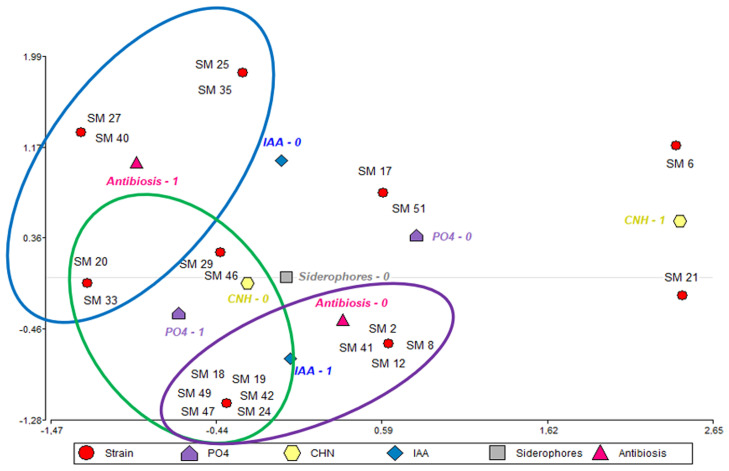
Correspondence analysis was constructed with variables resulting from the PGPR characterization of strains isolated from the M. verticillata rhizosphere. PO_4_, CHN, IAA. The value 0 in the description of each variable indicates negative activity (-), while the value 1 indicates positive activity (+).

Correspondence Analysis (CA) enables the visualization of variate observations on planes, facilitating the identification of the strongest associations between the categories of qualitative variables. As shown in Figure 2, three distinct clusters were identified. The first cluster (pink) included strains SM 40, 27, 25, 35, 20, and 33, which were grouped based on their biocontrol effect through antibiosis against pathogenic fungi. The second cluster (green) comprised strains SM 18, 19, 20, 24, 29, 33, 42, 46, 47, and 49, known for their ability to solubilize phosphates. The third cluster (violet) consisted of strains SM 47, 49, 42, 18, 19, 24, 8, 2, 12, and 41, which are characterized by their production of IAA.

### 2.5. Quantitative Values of IAA Production 

The strains were selected for their PGPR abilities demonstrated in previous assays. Among these strains, SM 33 exhibited the highest production, with a concentration of 936.3 ng/mL (Table 4). In concordance, Pedraza et al. [50] found that *P. stutzeri* strains produced IAA in a concentration ranging from 1000 to 2900 ng/mL protein in a culture medium supplemented with tryptophan. Due to its superior production capability, strain SM 33 was selected for subsequent plant inoculation assays.

### 2.6. Genotypic Identification

To determine the identity and phylogenetic position of the native strains isolated from the *M. verticillata* rhizosphere, nucleotide sequences of the 16S rRNA gene were analyzed. For this analysis, strains were selected based on their plant growth-promoting activities demonstrated in the previous assays. The chosen strains were SM 8, SM 20, SM 21, and SM 33, selected for their PGPR abilities to solubilize phosphates, produce IAA, and exhibit higher percentages of biocontrol through antibiosis. The 16S rRNA sequences of the strains under study were blasted with available sequences into the NCBI database. The homology analysis of the native strains from the *M. verticillata* rhizosphere (Table 5) shows that strains SM 21 and SM 33 share identity levels of around 0.99 for the 16S rRNA gene region with reference sequences such as *Pseudomonas putida* IR7-6 (MW686888.1) and *Stutzerimonas stutzeri* KGS-2 (CP018050.1), respectively. On the other hand, strains SM 8 and SM 20 had around 99% identity with *Pseudomonas* sp. However, the coverage value is insufficient to provide an accession number in NCBI GenBank (low similarity), as it does not meet the necessary coverage standards for reliable and complete taxonomic identification. The low percentage of coverage prevents the verification and formal registration of the sequence.

**Figure 3:**
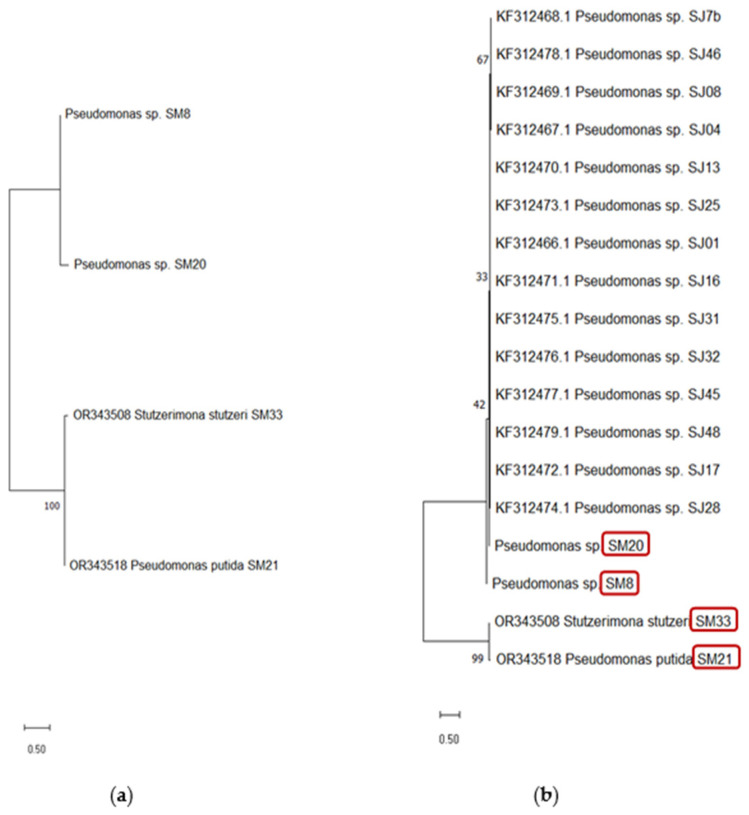
Phylogenetic tree of partial 16S rDNA sequences of plant-growth promoting strain: (**a**) four bacterial strains isolated from *M. verticillata* and (**b**) 14 bacterial strains isolated from the *M. piperita* rhizosphere and the strains isolated from *M. verticillata* (red frame). The numbers at the nodes indicate the bootstrap values out of 1000 replicates. The bar represents 0.50 substitutions per site.

Additionally, a phylogenetic tree was obtained (Figure 3), constructed with the 16S rRNA gene nucleotide sequences from the strains isolated from the *M. verticillata* rhizosphere (Figure 3a), and another considering the previous strains and the strains isolated from the *Mentha piperita* rhizosphere [41] (Figure 3b), with the aim of studying and comparing the evolutionary and phylogenetic relationships between the different microbial strains present in the rhizosphere of these two medicinal and aromatic plant species. The phylogenetic tree constructed (Figure 3a) involving four nucleotide sequences of the 16S gene showed that *Pseudomonas* sp. SM 8 is phylogenetically associated with *Pseudomonas* sp. 20, supported by a bootstrap value of 87. Likewise, in Figure 3b, it can be observed that SM 8 and SM 20 strains group into a cluster distinct from 21 and SM 21 strains, indicating that these strains may be phylogenetically closer to *Pseudomonas* sp. SJ 48 isolated from the mint rhizosphere.

### 2.7. Growth-Promoting Effects

**Figure 4:**
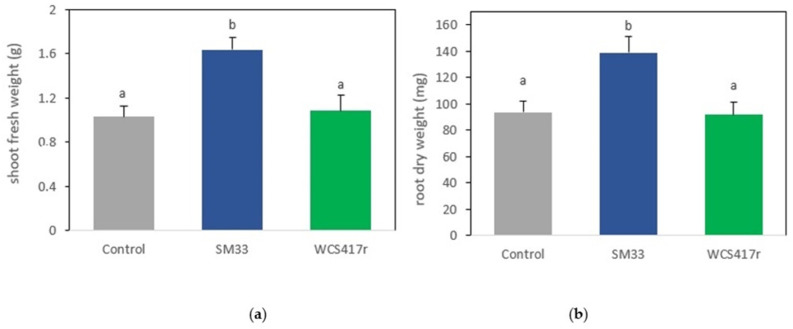
Effect of direct inoculation of *M. verticillata* with PGPR strains (SM 33 and WCS417r) on growth parameters: (**a**) Shoot fresh weight and (**b**) root dry weight. Different letters indicate significant differences. Letters above bars indicate significant differences according to Fisher’s LSD test (*p* < 0.05).

PGPR strain can stimulate plant growth through various mechanisms, including the production of substances that act as plant growth regulators or the stimulation of their biosynthesis by plants, improved nutrient assimilation, and the inhibition of fungal pathogens [51,52,53,54]. For this analysis, the isolated strain SM 33 was selected based on its plant growth-promoting activities, including phosphate solubilization and IAA production. In the present study, inoculation with the native strain SM 33 positively and significantly modified the evaluated growth parameters such as the number of leaves, number of branches, number of nodes, stem length, fresh shoot weight (Table 6), and dry root weight (*p* < 0.05) (Figure 4). This is consistent with data previously reported by our laboratory in *M. piperita* plants inoculated with PGPR strains of interest [55] and by numerous authors on different plant species belonging to the Lamiaceae family, such as basil, winter savory (*Satureja montana*), rosemary, among others [27,56,57,58,59,60,61].

The observed increase in all parameters, as a result of inoculation with PGPR strains, may be linked to the growth-promoting mechanisms present in these microorganisms. Specifically, in the case of strain SM 33, this growth enhancement could be related to its ability to produce IAA (Table 4). Although the strain WCS417r is considered a PGPR and various authors have found significant effects with its inoculation [62,63,64,65], our study did not show significant differences in the evaluated parameters compared to the control. These contradictory results indicate that the impact of PGPR application on cultivated plants is species and strain-specific [41,66].

In response to the direct inoculation of *M. verticillata* plants with PGPR strains, statistically significant differences (*p* < 0.05) were only evident in the shoot fresh weight when the plant was inoculated with the SM 33 strain (Figure 4a). The aerial fresh weight of inoculated plants was 160% and 129% higher in plants treated with the SM 33 strain compared to plants inoculated with WCS417r or uninoculated, respectively. Additionally, the root dry weight was 48% higher than the control. This increase in root weight is a common response to bacterial IAA production, as inoculation with IAA-producing bacteria induces the proliferation of lateral roots and root hairs [67]. Therefore, the observed increase in root weight may be attributed to the high levels of IAA produced by SM 33. Similarly, Chandra et al., [68] observed an increase in root and shoot biomass in crop plants as a result of inoculation with IAA-producing bacteria isolated from *Stevia rebaudiana*. Additionally, Yousef [69] demonstrated that IAA and PGPR significantly enhanced germination rates, root growth, and shoot growth in plants.

IAA enhances root length by increasing the number of root branches, root hairs, and root laterals that aid in the uptake of nutrients from the surroundings [68]. Synthesizing IAA is considered an effective tool for screening beneficial microorganisms, suggesting that IAA-producing bacteria have a profound effect on plant growth [70]. 

SM 33 was identified as *Stutzerimonas stutzeri* (formerly *Pseudomonas stutzeri*) [35] according to the study conducted on the 16S rRNA gene region, as previously mentioned (Table 4). Similar to our studies, it has been observed that the *P. stutzeri* strain possesses plant growth-promoting activity. For instance, *P. stutzeri* MJL19 stimulates the growth of soybean plants under saline stress conditions and shows excellent colonization efficiency, likely due to its strong chemotactic response to root exudates and enhanced biofilm formation capacity in the presence of high salt concentrations [36]. Additionally, another study found that inoculation with a certain concentration of *P. stutzeri* significantly promoted tomato growth and induced significant changes in tomato root exudates, which in turn significantly induced growth, swarming motility, and biofilm formation [71]. Furthermore, a nitrogen-fixing *P. stutzeri* strain, A15, isolated from the rice rhizosphere and endosphere, has been shown to promote plant growth as well [72].

## 3. Materials and Methods

### 3.1. Soil Sample Collection and Strain Isolation

*M. verticillata* plants grown in the Santa Maria de Punilla, Punilla department, Córdoba province, Argentina (31°44′43.9″ S 64°22′01.0″ W) were collected. The sample was collected with prior authorization for scientific collection and the use of said biological material by the Secretary of Environment of the Province of Córdoba (GOBDIGI-0158493111-522) in accordance with the Nagoya Protocol. Samples were stored at 4 °C until they were used for isolation of rhizobacteria. Roots were washed in sterile distilled water to remove loosely adherent soil and then rotary shaken for 20 min in 90 mL sterile phosphate buffer, pH 7, to obtain a rhizospheric soil suspension. Serial dilutions were prepared from the suspension. Then, 0.1 mL of each dilution was spread on Nutrient Agar medium (Britania, Buenos Aires, Argentina) and on King B medium [73] and incubated at 28 °C for 48 h. After incubation, total bacteria and fluorescent bacteria under UV light were counted as colony-forming units (CFU) per g rhizospheric soil [74]. Bacterial strains were purified on King B medium, identified, and characterized. After 48 h, bacterial counts were conducted on the dilutions that presented between 30 and 300 CFU. A collection of 44 native strains was generated from King B plates based on fluorescence, color, size, and colony morphology. The selected colonies were re-cultured on KB Agar until a pure culture was obtained. Subsequently, a Gram test using the KOH method was performed on each strain, and only those with Gram-negative characteristics were selected for further tests [63].

### 3.2. Determination of Physical and Chemical Properties of the Rhizosphere Soil

Physico-chemical analyses such as pH, total organic carbon, and total nitrogen were performed at the Laboratorio Integral Agropecuario in Río Cuarto, Córdoba, Argentina, using standard protocols. For this experiment, soil samples were analyzed for organic matter (OM) using the [75] method and for electrical conductivity (EC) with a 1:5 soil-to-water ratio following APHA (1998) guidelines. Nitrogen, in the form of nitrate ions (NO3-N), was extracted from the soil with water and quantified colorimetrically after reacting with phenol sulfonic acid. Potassium content was determined by extracting the soil with Bray’s extracting reagent and measuring available potassium using an absorptiometer [76]. Soil pH was measured using a digital pH meter (Mettler-Toledo, Cologne, Germany), and soil texture was determined according to [77].

### 3.3. Phenotypic Characterization

Native strains were phenotypically characterized based on morphological, physiological, and biochemical criteria [46,78,79]. The tests and parameters included colony morphology, Gram determination using KOH test [80], catalase determination [81], oxidase determination (Britania), fluorescence color under UV illumination, pigment production [73], temperature tolerance, growth at 5% and 6.5% salt concentration, Tween 80 hydrolysis [82], starch hydrolysis [83], casein hydrolysis [84] and lecithinase production [85]. Each test was performed twice.

### 3.4. Determination of the Main Direct and Indirect Mechanisms of Plant Promotion

#### 3.4.1. Indole-3-Acetic Acid Production

##### Qualitative Determination

Indole-3-acetic acid (IAA) level was qualitatively assayed as described in [86]. The test was performed both in the presence and absence of L-tryptophan as a precursor of IAA. One hundred mL LB broth in 250 mL Erlenmeyer flasks were inoculated with 100 μL of overnight bacterial culture, adjusted to optical density 0.5 (10^7^–10^8^ CFU mL^−1^) measured at 600 nm. The bacteria were grown at 28 ± 2 °C for 96 h with continuous shaking at 150 rpm. The supernatant was collected by centrifuging at 10,000 rpm for 10 min. IAA was detected by mixing 100 μL of Salkowski reagent (1 mL 0.5 M FeCl_3_, 30 mL concentrated H_2_SO_4_, and 50 mL distilled H_2_O) with 100 μL supernatant and allowed to react at room temperature for 30 min. IAA production was confirmed by pink color development. Blanks (LB broth medium and Salkowski reagent) showing yellow color were below the detection limit. *Azospirillum brasilense* Sp7 was used as a positive control. The assay was replicated twice.

##### Quantitative Determination

As described in [87], the identification and quantification of the analyte under study was carried out using liquid chromatography coupled with tandem mass spectrometry (LC-MS/MS) for four selected strains isolated from the *M. verticillata* rhizosphere. The liquid chromatograph used was Alliance 2695 (Waters Inc., San Jose, CA, USA), and chromatographic conditions were gradient with methanol (100%): water/acetic acid (0.2%) (20:80) at a flow rate of 0.2 mL/min. Subsequently, they were analyzed by a double mass spectrometer coupled with Quatro UltimaTM Pt (Micromass, Manchester, UK) through the MassLynk software v4.0.

#### 3.4.2. Siderophore Production

Siderophore production was qualitatively assayed using the chrome azurol S (CAS) method with some modification [88]. Petri dishes were coated with 15 mL M9 medium. After the medium solidified, one-half was cut off and replaced by 7.5 mL CAS-blue agar [89]. Bacteria were inoculated in the boundary between the two media. A CAS-agar plate was inoculated with *Sinorhizobium meliloti* strain Rm1021 as a negative control [89]. Plates were incubated for 48 h at 28 °C. CAS reaction was determined by a color change from blue to orange. The lack of siderophore production by the negative control was evidenced by the absence of an orange color around the colonies. The technique was validated using color development by colonies of the native fluorescent strains. The assay was carried out twice.

#### 3.4.3. Phosphate Solubilization Ability

Phosphate solubilization ability was qualitatively assayed by inoculating bacteria as a single point on Petri dishes coated with Pikovskaya medium [90]. Plates were incubated for 48 h at 28 °C. The formation of a clear zone around bacterial colonies was considered to be a positive reaction. *S. meliloti* RM1021 was used as a negative control. The lack of phosphate solubilization in the negative control was evidenced by the absence of a clear zone around the colonies. The technique was validated as the formation of clear zones around colonies of the native fluorescent strains. The assay was replicated twice.

#### 3.4.4. Hydrogen Cyanide (HCN) Production

The bacterial strains to be tested were streaked onto Petri dishes containing 10% diluted TSA supplemented with the amino acid glycine (4.4 g/L). Filter paper impregnated with 0.5% picric acid and 2% sodium carbonate was placed on the inner side of the Petri dish lid. The plates were then incubated at 28 °C for 3 days. Plates where the filter paper changed color from yellow to orange-brown are considered to be HCN producers [91].

#### 3.4.5. Biocontrol by In Vitro Antibiosis

The ability of bacterial strains to inhibit the growth of the phytopathogenic fungus *Sclerotium rolfsii* was assessed using a method described by [92]. The bacterial strains were streaked onto 25% Trypticase Soy Agar (TSA) plates, covering one-quarter of the plate. After 24 h of incubation, a fungal plug of *S. rolfsii* was placed on the opposite side of the plate. The plates were then incubated for 4 days. The results were expressed as the percentage of fungal growth inhibition compared to a control. The percentage of mycelial growth inhibition was calculated by subtracting the percentage of mycelial growth in the interaction zone from 100%.

### 3.5. Amplification and Bioinformatic Analysis of 16S rRNA Region Sequences

#### 3.5.1. DNA Extraction

A single colony was inoculated on 3 mL LB medium and incubated at 28 °C for 24 h. A 2-mL culture sample taken at a late exponential growth phase was subjected to DNA extraction using a Genomic DNA Purification Kit (Fermentas, Thermo Fisher Scientific; Pittsburgh, PA, USA) according to the manufacturer’s instructions.

#### 3.5.2. 16S rRNA Gene Nucleotide Sequence Analysis

Four strains isolated from the rhizosphere of *M. verticillata* cultivation were selected for their characteristics of promoting plant growth, and the corresponding analysis of the 16S rRNA gene sequences was performed. Direct PCR was performed with 1 µL DNA template in a 20-µL PCR reaction mixture containing universal primers 27F(5′AGAGTTTGATCCTGGCTCAG3′), 1492R(5′TACGGTTACCTTGTTACGACTT-3′), 518F (5′-CCAGCAGCCGCGGTAATACG-3′) y 800R (5′-TACCAGGGTATCTAATCC-3′) [93]. Purified PCR products (~1400 bp) were sequenced by Macrogen, Inc. (Seoul, Korea) using an automated DNA sequencing system (model 3730XL, Applied Biosystems; Foster City, CA, USA). The obtained sequences were compared with deposited sequences in to NCBI public database using the BLASTn search program [94]. GeneBank accession numbers for the 16S rRNA sequences for each analyzed strain were obtained when high homology and coverage were found.

#### 3.5.3. Construction of the Phylogenetic Tree

Two phylogenetic trees were constructed: one with the sequences obtained from the four selected strains isolated from *M. verticillata* and another tree including these sequences and those obtained from GenBank of 14 strains previously isolated by Santoro et al. [41]. from the rhizosphere of *Mentha piperita* (*Pseudomonas* sp. SJ01 accession number (No.): KF312466.1, *Pseudomonas* sp. SJ04 accession number (No.): KF312467.1, *Pseudomonas* sp. SJ7b accession number (No.): KF312468.1, *Pseudomonas* sp. SJ08 accession number (No.): KF312469.1, *Pseudomonas* sp. SJ13 accession number (No.): KF312470.1, *Pseudomonas* sp. SJ16 accession number (No.): KF312471.1, *Pseudomonas* sp. SJ17 accession number (No.): KF312472.1, *Pseudomonas* sp. SJ25 accession number (No.): KF312473.1, *Pseudomonas* sp. SJ28 accession number (No.): KF312474.1, *Pseudomonas* sp. SJ31 accession number (No.): KF312475.1, *Pseudomonas* sp. SJ32 accession number (No.): KF312476.1, *Pseudomonas* sp. SJ45 accession number (No.): KF312477.1, *Pseudomonas* sp. SJ46 accession number (No.): KF312478.1, *Pseudomonas* sp. SJ48 accession number (No.): KF312479.1).

Sequence alignment was performed using Clustal W with BioEdit version 7.2.5 [95], and the evolutionary analyses were conducted in MEGA11 [95]. The phylogenetic trees were inferred using the Neighbor-Joining method [96]. The percentage of replicate trees in which the associated taxa clustered together in the bootstrap test (1000 replicates) are shown next to the branches [97]. Only values greater than 50% are indicated. The tree is drawn to scale, with branch lengths in the same units as those of the evolutionary distances used to infer the phylogenetic tree (scale bar). The evolutionary distances are the difference in nucleotide base and were computed using the using the Kimura 2-parameter method [95] and are in the units of the number of base substitutions per site. The rate variation among sites was modeled with a gamma distribution (shape parameter = 1).

### 3.6. Evaluation of Growth-Promoting Effects

To evaluate the effects of *M. verticillata* seedling inoculation, the methodology of [5] was used with some modifications. *M. verticillata* seedlings with three growth nodes were transplanted into pots with soil mixed with perlite (80–20, *v*/*v*) and inoculated at the root crown with the isolated strain SM 33, selected for its PGPR characteristics, or *Pseudomonas simiae* WCS417r, used as a reference strain due to its PGPR characteristics demonstrated in previous studies [41,55]. Prior to inoculation, bacterial cultures were grown overnight at 28 °C with rotation at 120 rpm until reaching the exponential phase. Each culture was then washed twice in 0.9% NaCl by centrifugation (10,000 rpm, 10 min) in an Eppendorf centrifuge, resuspended in physiological solution, and adjusted to a final concentration of ∼10^9^ colony-forming units (CFU)/mL for use as an inoculum. Saturated cultures were diluted in sterile distilled water to a final concentration of 10^9^ CFU mL^−1^. Seedlings were inoculated with 100 μL of the corresponding bacterial suspension, while control plants were inoculated with 100 μL of sterile physiological solution.

After inoculation, plants were grown in a growth chamber with controlled conditions of light (16/8 h light/dark cycle), temperature (24 ± 2 °C), and relative humidity (∼70%). All plants received distilled water (20 mL/pot) once per week [55]. The experiments were performed under non-sterile conditions. The experiments were replicated (10 pots per treatment; 1 plant per pot), and the pots were arranged randomly in the growth chamber. Forty-five days after inoculation, the plants were removed from the pots, the roots were washed to remove soil, and standard growth parameters (shoot length, leaf number, node number, shoot fresh weight, root dry weight) were measured [55].

### 3.7. Statistical Analysis

Plant data were subjected to analysis of variance (ANOVA) followed by a comparison of multiple treatment levels with the control using the post hoc Fisher Least Significant Difference (LSD) test. Additionally, a multivariate cluster analysis was conducted to observe relationships between the isolated native strains and the determined biochemical variables, and a Correspondence Analysis (CA) was performed to observe associations with growth promotion variables. Infostat software program 2020 (Group Infostat, Universidad Nacional de Córdoba, Argentina) was used for all statistical analyses.

## 4. Conclusions

This study successfully isolated and characterized native *Pseudomonas* strains from the rhizospheric soil of *Minthostachys verticillata* plants, demonstrating their potential as plant growth-promoting rhizobacteria (PGPR). Through a series of biochemical tests, the strains exhibited capabilities such as phosphate solubilization and hydrogen cyanide production alongside biocontrol properties. The strain SM 33, which has a high similarity to *Stutzerimonas stutzeri* based on 16S rRNA gene sequencing, demonstrated significant growth-promoting effects on *M. verticillata* seedlings, increasing both shoot fresh weight and root dry weight of the plants. Future studies will require complete genome sequencing to ensure accurate identification and to fully characterize the strain. These findings underscore the potential application of native *Pseudomonas* strains in enhancing plant growth and health, offering promising avenues for sustainable agricultural practices.

## Figures and Tables

**Table 1:** Chemical characteristics of rhizosphere soil from *M. verticillatta* plants.

Chemical Characteristics	
pH	7.50
OM (%)	5.48
H° (%)	32.10
P (ppm)	57.00
N-NO_3_ (ppm)	23.30
NO_3_ (ppm)	103.22
Ca^+2^ (cmol/Kg)	16.00
Mg^+2^ (cmol/Kg)	6.50
Na^+^ (cmol/Kg)	0.90
K^+^ (cmol/Kg)	2.31
CEC (cmol/Kg)	27.83

**Table 2:** Phenotypic characterization of native *Pseudomonas* strains, based on morphological, physiological, and biochemical evaluation of native strains isolated from the *M. verticillata* rhizosphere. ‘+’ indicates presence, ‘−’ indicates absence, and ‘+/−’ indicates variable presence.

Strain	Gram	Catalase	Oxidase	Pigmentation	Growth				Hydrolysis			
				King B	4 °C	42 °C	NaCl 5%	NaCl 6.5%	Lipids	Starch	Casein	Lecithinase
SM 2	−	+	+	green	−	−	+/−	−		−	−	−
SM 6	−	+	+	orange	−	−	−	−	+	−	+	−
SM 8	−	+	+	green	−	−	+/−	−	−	−	−	−
SM 12	−	+	+	orange	−	−	−	−		−	−	−
SM 17	−	+	−		−	−	−	−	+	−	+	−
SM 18	−	+	−	orange	−	−	+/−	−	−	−	−	−
SM 19	−	+	−	orange	−	−	+/−	−	−	−	−	−
SM 20	−	+	+	green	+	−	−	−	−	−	+	−
SM 21	−	+	+	green	+	−	−	−	−	−	+	−
SM 24	−	−	+		−	−	−	−	−	−	−	−
SM 25	−	+	−		−	+	−	−	+	−	−	+
SM 27	−	+	+	green	−	−	−	−	−	−	−	−
SM 29	−	+	+		+	−	−	−	−	−	−	−
SM 33	−	+	+		+	−	+/−	−	−	−	−	−
SM 35	−	+	+	green	−	+	−	−	−	−	−	−
SM 40	−	+	+		+	+	+	−	+	+	+	+
SM 41	−	+	+		+	−	+	−	−	−	+	−
SM 42	−	+	+		+	−	−	−	−	−	+	−
SM 46	−	+	+	green	−	−	+/−	−	−	−	−	−
SM 47	−	+	+	green	+	−	−	−	−	−	−	−
SM 49	−	+	+		+	−	−	−	−	−	+	−
SM 51	−	+	+		+	+	−	−	+	+	−	−

**Table 3:** PGPR activity in native strains isolated from the *M. verticillata* rhizosphere. ‘+’ indicates presence, ‘−’ indicates absence.

Strain	Phosphate Solubilization	Siderophores Production	IAA Production	CHN Production	Antibiosis %
SM2	−	−	+	−	19
SM6	−	−	−	+	5
SM8	−	−	+	−	21
SM12	−	−	+	−	5
SM17	−	−	−	−	0
SM18	+	−	+	−	12
SM19	+	−	+	−	9
SM20	+	−	+	−	31
SM21	−	−	+	+	14
SM24	+	−	+	−	14
SM25	−	−	−	−	50
SM27	+	−	−	−	29
SM29	+	−	−	−	19
SM33	+	−	+	−	34
SM35	−	−	−	−	38
SM40	+	−	−	−	33
SM41	−	−	+	−	14
SM42	+	−	+	−	12
SM46	+	−	−	−	14
SM47	+	−	+	−	19
SM49	+	−	+	−	19
SM51	−	−	−	−	19

**Table 4:** Quantification of IAA by LC-MS/MS Method produced by strains isolated from the *M. verticillata* rhizosphere.

Sample	IAA (ng/mL)
Control LB	30.9
SM 8	556.7
SM 20	578.1
SM 21	717.8
SM 33	936.3

**Table 5:** Data from BLASTn analysis of 16S rRNA gene sequences from native strains isolated from *M. verticillata* rhizosphere.

Native Strain	Aligned Species (Access Number)	Homology	Coverage	Annotation (Access Number)
SM 8	*Pseudomonas* sp.	99%	~61–63%	*Pseudomonas* sp.
SM 21	*Pseudomona putida* IR7-6 (MW686888.1)	99%	99%	*Pseudomonas putida* SM21 (OR343518)
SM 33	*Stutzerimona stutzeri* KGS-2 (CP018050.1)	99%	95%	*Stutzerimona stutzeri* SM33 (OR343508)
SM 20	*Pseudomonas* sp.	99%	~62–63%	*Pseudomonas* sp.

**Table 6:** Effect of direct inoculation of native strain SM33 on plant growth parameters of *M. verticillata*. Data shown are means ± SE. Values within a column followed by the same letter are not significantly different according to Fisher’s LSD test (*p* < 0.05).

	Leaf Number	Ramification Number	Node Number	Shoot Lenght (cm)
control	17.50 ± 1.20 a	0.5 0± 0.13 a	7.17 ± 0.22 a	15.67 ± 0.99 a
SM 33	24.07 ± 1.12 b	1.70 ± 0.23 b	8.30 ± 0.22 b	20.07 ± 0.83 b
WCS417r	17.27 ± 1.33 a	0.85 ± 0.21 a	7.00 ± 0.18 a	15.75 ± 1.15 a

## Data Availability

The raw data supporting the conclusions of this article will be made available by the authors upon request.
